# Maleic and l-tartaric acids as new anti-sprouting agents for potatoes during storage in comparison to other efficient sprout suppressants

**DOI:** 10.1038/s41598-021-99187-y

**Published:** 2021-10-08

**Authors:** Ekta Bhattacharya, Suparna Mandal Biswas, Panchanan Pramanik

**Affiliations:** 1 Agricultural and Ecological Research Unit, Indian Statistical Institute, 203, B.T. Road, Calcutta, 700108 India; 2 Department of Chemistry, GLA University, Mathura, 281406 India

**Keywords:** Biochemistry, Chemical biology, Plant sciences

## Abstract

Inhibiting sprouting of potatoes is an interesting subject needed for potato storage and industry. Sprouting degrades the quality of tuber along with releasing α-solanine and α-chaconine, which are harmful for health. Sprout suppressants, available in the market, are either costly or toxic to both health and environment. So, there is a need for developing countries to explore new sprouting suppressant compound which is cheap, non-toxic and reasonably efficient in comparison to commercial ones. We have established that simple maleic acid and l-tartaric acid are effective sprout suppressing agents. Both can hinder sprouting up to 6 weeks and 4 weeks post treatment respectively at room temperature in dark. These do not affect the quality parameters, retain the moisture content and maintain the stout appearance of the tubers along the total storage period. Thus maleic acid and l-tartaric acid would qualify as alternative, cheap, efficient sprout suppressant for potato storage and processing.

## Introduction

Potato (*Solanum tuberosum* L.) ranked fourth as the world’s main food crop, following maize, wheat, and rice^1^. Being the world’s number one non-grain food crop, its shelf-life is of great concern. Potatoes are generally stored at a low temperature for several months as a measure to delay sprouting^2^. Based on the geographical region, fresh potatoes are available only for a few months. Thus storage of potatoes is necessary to maintain supply throughout the year. Germination and growth of the eyes of potatoes, is a significant factor that contributes towards the weight loss of the potato and facilitates production of toxic α-solanine and α-chaconine with symptoms ranging from nausea, vomiting, diarrhea, and fever to delirium, coma, and even death^3,4^. It also alters the taste of the potato. Upon sprouting, respiration as well as transpiration increase rapidly which in turn increases the rate of physiological weight loss of stored tubers. Besides weight loss, sprouting also affects the nutritional values and quality of potatoes^5,6^. Sprouting causes higher rate of respiration, remobilization of storage compounds in the potato tubers mainly starch and proteins, besides shrinkage, due to loss of water^7^. Higher level of reducing sugar content also causes lower processing quality of potato tubers^8^. It also increases sugar concentrations through hydrolysis^9,10^. Sprouting also denatures potato quality parameters such as firmness and content of vitamin C^11^.

The most widely used sprout suppressant on potatoes all over the world for more than 50 years is CIPC [isopropyl *N*-(3-chlorophenyl) carbamate]^12^. The cost of CIPC treatment per kg of potato in cold storage houses ranges from 0.14 to 0.54 INR^13^. But continuous and long term use of CIPC leads to some toxicological effects on health and environment. It was reported that up to 45% of applied CIPC is persistent in the soil besides adhering to tubers^14^. It was observed that the CIPC residue in peel samples were fairly high about 15–85 mg/kg^15,16^. CIPC renders sprout growth by blocking the spindle formation during cell division^17,18^. For that reason its uptake into the body can also cause alteration in cellular structure and functions. In addition, due to the low solubility in water (89 mg/l), organic solvents (like; methanol or dichloromethane) are needed for its application as a fogging treatment which not only creates pollution to the environment but also imposes the risk on the personnel involved in treating/fogging application^19^.

There are some reports of organic compounds those have been developed for antisprouting of potato tubers and are in practice. Aliphatic keto- and aldo-compounds like 3-decene-2-one, 3-decanone and trans-2-nonenal can suppress sprouting upto 17 and 12 weeks, respectively^20,21^. The main active compounds of essential oils like carvone, eugenol, cinnamaldehyde, thymol, citral, geraniol, and citronellol can also suppress sprouting of potatoes upto 2–5 weeks^22–24^. The compounds, structures and their comparative efficacies are listed in Table 1.

**Table 1:** List of reported compounds used for sprout suppression of potato tubers and their respective references.

Sl No	Name	Concentration	Activity	References	Structure
1	CIPC	17 ppm	20–24 weeks	^12^	
2	1,2-Dimethyl napthalene	20 ppm	12 weeks	^12^	
3	3-Decene-2-one (smart block)	Thermal fogging	8 weeks	^19^	
4	3-Decanone	0.75 mmol/kg tuber	12 weeks	^20^	
5	Trans-2- nonenal	0.5–1 mmol/kg tuber	17 weeks	^20^	
6	3-Nonen-2-one	0.75 mmol/kg tuber	3 weeks	^20^	
7	Trans-2-hexenal	0.6–4.3 mmol/kg	4 weeks	^20^	
8	Trans-2-hexenol	0.5–0.75 mmol/kg	3 weeks	^20^	
9	Carvone	Head space conc. 5–10 µg/l	As long as the carvone is applied. More than 4 weeks	^21^	
10	Salicaldehyde	3.6 × 10^–5^ M/head space	2 weeks	^22^	
11	Cinnamaldehyde	1.9 × 10 ^−6^ M	2 weeks	^22^	
12	Eugenol	10 ppm	2–5 Weeks	^22^	
13	Thymol	8 mM emulsion	1 week	^22^	
14	Citral	6–8 mM	16 weeks	^23^	
15	Geraniol	6–8 mM	16 weeks	^23^	
16	Citronellol	6–8 mM	2 weeks	^23^	
17	Maleic hydrazide	20–50 ppm	4 weeks	^24^	

But these natural compounds have some demerits too like cost, large scale commercial unavailability. So, there is still a need to find new sprout suppressant of potatoes that would be natural, effective, cheap and safe alternative^25^. Potatoes require normally 4 weeks or more to reach consumers from cold storage facilities after harvesting. So antisprouting agent should have activity at least 4 weeks or more.

The effect of maleic hydrazide (MH) on sprout suppression of potatoes have been reported and thoroughly studied by various researchers^26–29^. But studies have also reported that MH possesses cytotoxic activities and leaves residues on treated potato tubers^30,31^. However, in this study we focused on the antisprouting mechanism of MH where it releases maleic acid after oxidation^32^. This provided a clue that maleic acid and similar types of organic acids may have some role as antisprouting agents of potatoes.

Therefore, the objective of the present study was to evaluate the efficacy of various organic acids (namely l-tartaric acid, maleic acid, d-tartaric acid, oleic acid, palmitic acid, stearic acid, fumaric acid, malic acid) and aromatic acids (namely salicylic acid, 4-hydroxy benzoic acid, 2-hydroxy cinnamic acid, ferrulic acid, caffeic acid, gallic acid)for controlling potato tuber sprouting during open storage and their impact on loss of fresh weight, reducing sugar content, total protein content, total phenolic content in potato tubers.

## Results

### Sprout inhibition assay

**Figure 1:**
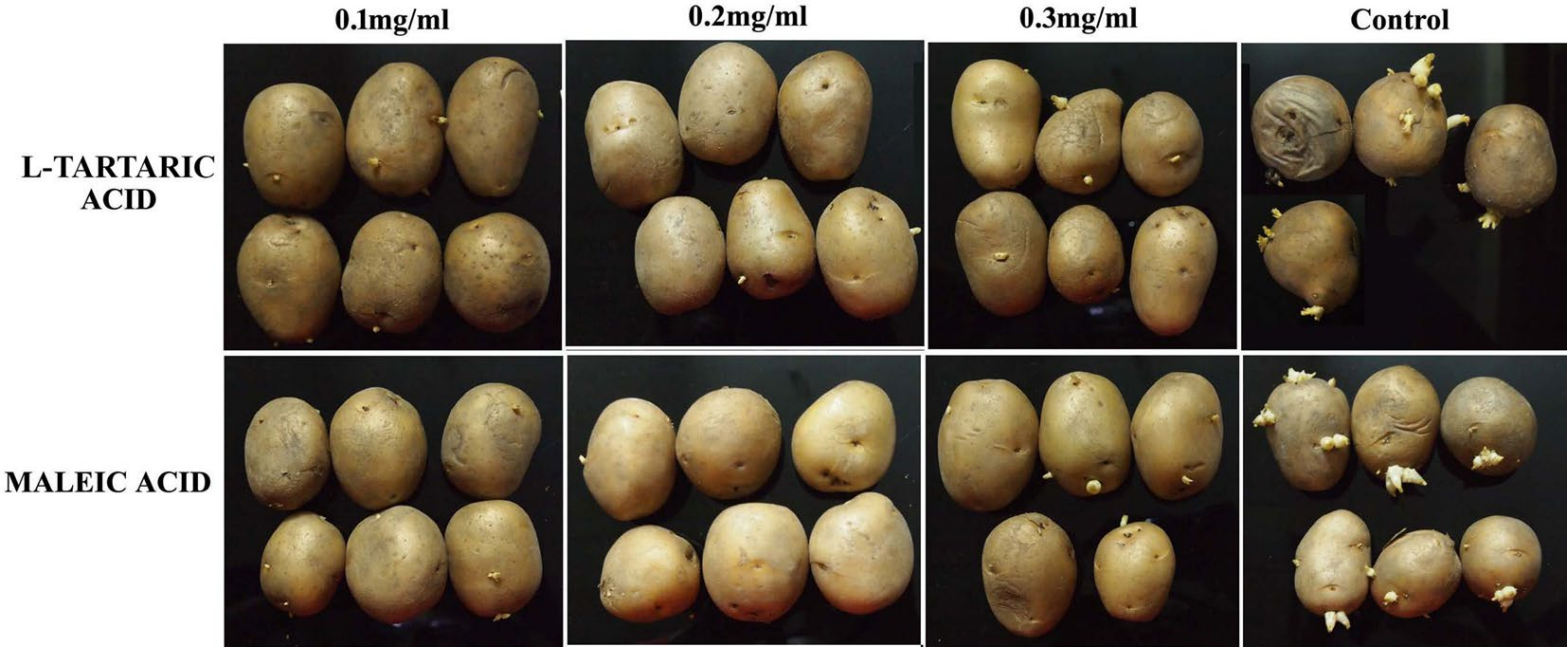
Effect of maleic acid and l-tartaric acid at different concentrations (ranging from 0.1 to 0.3 mg/ml) on potato tubers after 6 weeks of storage at room temperature in dark.

It was observed that sprouting was stimulated when the tubers were exposed to light. Therefore, after treatment, the tubers were stored in dark at room temperature. A preliminary experiment was performed with the mentioned compounds (Table 2). Compounds that could check sprouting of potatoes even after the control set had sprouted above 95% were considered as a marker for selecting the compounds that show promise against sprouting of potato. Among all the tested compounds, maleic acid and l-tartaric acid revealed satisfactory results in hindering sprouting of potato tubers (Fig. 1). Therefore, detailed experiments for estimating the quality parameters after the treatment and storage period along with antisprouting efficiency were done using these two compounds (Supplementary information). The average sprouting percentage for maleic acid at 0.1, 0.2 and 0.3 mg/ml concentration was 23.81, 2.5 and 9.12 respectively after 42 days post treatment. While, for that of l-tartaric acid, it was 35.92, 12.97, 18.59 respectively post treatment (Table 3). The data was first tested for normality using Kruskal–Wallis test. Upon testing, a two way ANOVA was conducted to compare the main effects of concentrations and time of incubation for maleic acid and l-tartaric acid as well as their interaction effects on the sprouting percentages.

**Table 2:** Sprouting inhibition efficiency of various organic acids (namely l-tartaric acid, maleic acid, d-tartaric acid, oleic acid, palmitic acid, stearic acid, fumaric acid, malic acid) and aromatic acids (namely salicylic acid, 4-hydroxy benzoic acid, 2-hydroxy cinnamic acid, ferrulic acid, caffeic acid, gallic acid).

Sl no.	Compounds studied in this investigation	Concentration (mg/ml)	Activity	Structure
1	Maleic acid	0.2-0.5	6 weeks	
2	Fumaric acid	0.2-0.5	No activity	
3	Malic acid	0.2-0.5	No activity	
4	Meso-tartaric acid	0.2-0.5	No activity	
5	l (+) tartaric acid	0.2-0.5	4 weeks	
6	d (−) Tarataric acid	0.2-0.5	No activity	
7	Oleic acid	0.2-0.5	No activity	
8	Palmitic acid	0.2-0.5	No activity	
9	Stearic acid	0.2-0.5	No activity	
10	Salicylic acid	0.2-0.5	No activity	
11	4-Hydroxy benzoic acid	0.2-0.5	No activity	
12	2-Hydroxy cinnamic acid	0.2-0.5	No activity	
13	4-Hydroxy cinnamic acid	0.2-0.5	No activity	
14	Ferrulic acid	0.2-0.5	1–2 weeks	
15	Caffeic acid	0.2-0.5	1–2 weeks	
16	Gallic acid	0.2-0.5	1–2 weeks	

Concentration tested are 0.2, 0.3 and 0.5mg/ml.

**Table 3:** Sprouting percentage data of the control and treated potato tubers after the first 7 days and at the end of the 42 days storage period post treatment.

Treatments concentrations (mg/ml)	Sprouting percentages			
	Maleic acid		l-Tartaric acid	
	7 days	42 days	7 days	42 days
0	78.86 ± 19.69^a^	98.07 ± 4.38^a^	63.3 ± 13.89^a^	98.4 ± 3.93^a^
0.1	23.81 ± 11.93^b^	33.41 ± 15.39^b^	13.9 ± 14.47^b^	35.92 ± 7.44^b^
0.2	2.5 ± 5.05^c^	7.48 ± 9.53^c^	4.76 ± 6.81^c^	12.97 ± 12.66^c^
0.3	9.12 ± 7.76^c^	11.84 ± 6.45^c^	5.15 ± 7.41^c^	18.59 ± 11.61^c^

The data is represented as mean ± SD. The values with different superscripts “a,b,c” are significantly different along the columns.

The concentration and time of incubation effects were statistically significant with p < 0.00. The main effect of concentration yielded an effect size of 0.878, indicating that 87.8% of the variance in the sprouting percentage can be strongly explained by concentration of the treatments [F (3,176) = 421.41, p = 0.00]. The main effect of time of incubation yielded an effect size of 0.210, indicating that 21.0% of the variance in the sprouting percentage was explained by time of incubation [F (3,176) = 15.67, p = 0.00]. The interaction effect was also significant [F(9,176) = 3.169, p = 0.01], indicating the combined effect of concentrations and time of incubation for both maleic acid and l-tartaric acid with an effect size of 0.139, i.e., 13.9% of the variance in the sprouting percentage can be explained by both the independent variables. According to the Tukey post hoc test, in terms of concentration, sprouting percentage did not vary significantly at 0.2 mg/ml and 0.3 mg/ml for both the treatments.

It was observed that the eyes on the potato tubers turned black after treatment with maleic acid. This observation is encouraging i terms of sprout suppression.

### Loss of fresh weight

**Figure 2:**
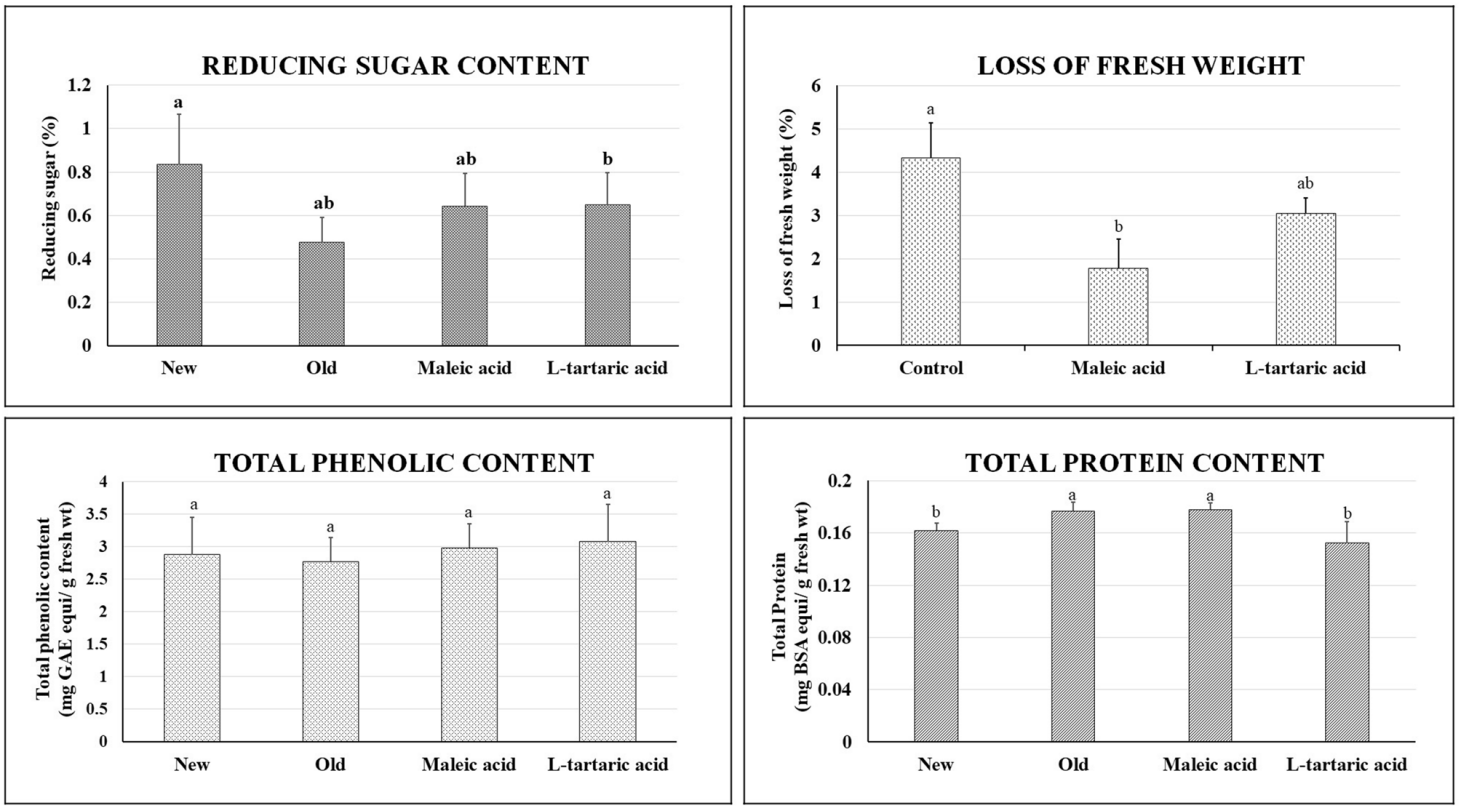
Quality parameters i.e. Loss of Fresh weight, Reducing sugar content, Total phenolic content, Total protein content after storage period of 42 days. “New” denotes the fresh tubers and “Old” denotes the untreated tubers post the storage period of 42 days. The data presented is the mean ± SD. The bars denoted by the same letters "a, b, c" are not significantly different.

Fresh weight loss was evident after storage period of 42 days. Initial and final weight was measured for all the sets. It was observed that the control set lost about 4.3% of initial fresh weight in the course of time due to sprouting and desiccation. Sprouting resulted in enhanced water loss through increased transpiration rate. In case of maleic acid, only 1.77% and for l-tartaric acid average of 3.04% loss of initial fresh weight was observed which was may be due to its sprout suppressant effect. The mean percentage of loss of fresh weight was significantly different as compared to control in case of maleic acid, but not for l-tartaric acid according to the Tukey post hoc test (Fig. 2).

### Reducing sugar content

Reducing sugar content was measured for fresh potatoes as well as treated and untreated potatoes after the storage period. It was seen that fresh potatoes contained significantly higher amount of reducing sugar about 0.83% of fresh weight as compared to the stored ones. After the storage period, all the sprouted as well as non-sprouted potatoes had undergone degradation in amounts of reducing sugar (Fig. 2). The reducing sugar percentage of fresh potatoes (0.83%) differed significantly with the untreated potatoes (0.474%), the l-tartaric acid treated potatoes (0.642%) and the maleic acid treated potatoes (0.68%).

### Total protein content

Total protein content was measured as mg BSA equivalent per g fresh weight. It was observed that there is no significant difference between the fresh and stored potatoes. Fresh untreated tubers contained 0.161 mg BSA equivalent/g fresh wt, while in case of old untreated tubers the total protein was recorded as 0.177 mg BSA equivalent/g fresh wt. This did not differ significantly among all the treatments. The protein content ranged from 0.152 to 0.177 mg BSA equivalent/g fresh wt (Fig. 2).

### Total phenolic content

Total phenolic content was measured as mg GAE equivalent per g fresh weight. In this case too, it was observed that no significant differences were present in phenolic contents with the new and stored tubers. The phenolic content ranged from 2.77 to 3.07 mg GAE equivalent/g fresh wt among the fresh, untreated as well as treated set of tubers that were not statistically different [F (3, 32) = 0.687, p = 0.567]. In fresh potato tubers, 2.87 mg GAE equivalent/g fresh wt was measured whereas in maleic and l-tartaric acid treated tubers, the amounts recorded were 2.98 mg and 3.07 mg GAE equivalent/g fresh wt (Fig. 2).

## Discussion:

Potato plays a vital role as tuber crop in the global food system especially in food and nutrition security, poverty alleviation, environmental conservation and sustainable development. So, it is an important concern for storing harvested potatoes timely with proper sprout suppressants. The widely used CIPC has been reported to have some toxic effects on health as well as on environment due to their degraded by-products, uptake and residual effect on the tubers, low volatilization and less water solubility^15,16,26,33^. Residual effects of CIPC is noticed not only in the stored potatoes but also in the processed potato products^34,35^. Besides CIPC, there are several reports of sprout suppressants derived from natural and synthetic compounds such as ethylene and 1-MCP (1-methylcyclopropene) as well as techniques like ultraviolet-C irradiation and fumigation using plant essential oils^36–39^. Table 1 provides the compounds documented by various scientists and their comparative efficacy as anti-sprouting agents.

**Figure 3:**
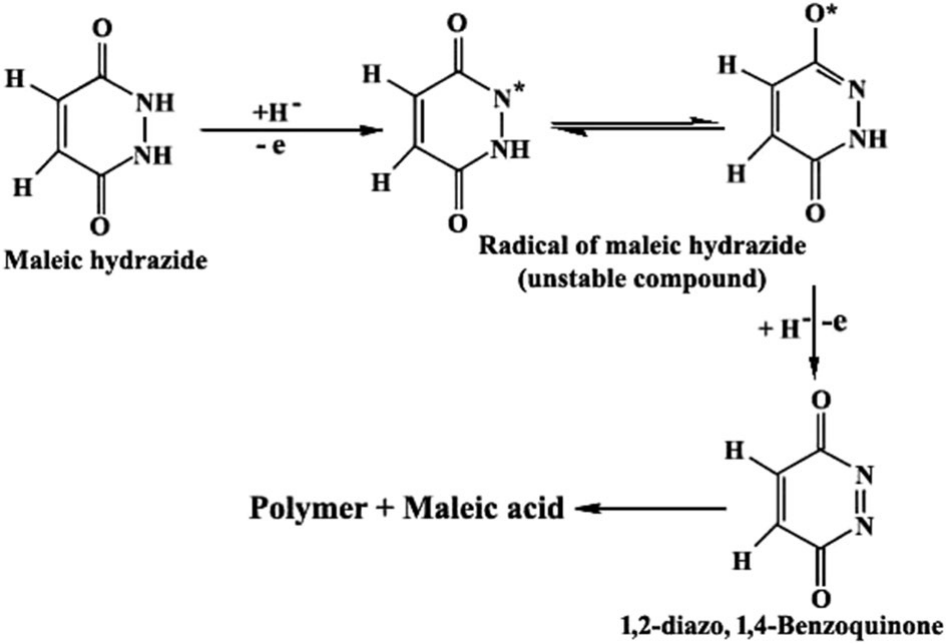
The tentative mechanism of activity of the antisprouting compounds.

Maleic hydrazide is a growth regulator and finds many applications in agriculture^26,27^. It is a well-known sprout suppressant but its mode of action is not clear. Through oxidation process by enzyme it releases N_2_ and maleic acid or some radicals which disrupt the structure of effective enzyme responsible for sprouting activity. We identified maleic acid during controlled oxidation of maleic hydrazide with Fenton’s reagent (Fe2^+^ with H_2_O_2_). This observation provoked us to test for antisprouting activity with maleic acid and structurally similar organic acids (Table 2). Interestingly, among the studied the activities of simple aliphatic and aromatic acids, we found that only maleic acid and l-tartaric acid have sufficient antisprouting activities for commercial application for storage of potatoes. Maleic acid was best, having activity for 6 weeks at room temperature in dark whereas l-tartaric was found to be active for 4 weeks. Tested aromatic organic acids (10–16 in Table 2) were not found effective except some phenolic acids namely; ferrulic, caffeic and gallic acid, which inhibited sprouting for 1–2 weeks.Tentative mechanism may be as follows (Fig. 3). Maleic acid contains acid group and an olefinic bond. This small molecule shows the antisprouting activity probably through some enzymes of the potato tuber. It is observed that other tested organic acids did not show any antisprouting properties. Simultaneously, fumaric acid which is the trans-isomer of maleic acid also does not show this unique biological activity. It may be presumed that maleic acid possesses right molecular conformation for docking with any suitable portion of the enzyme which causes the inhibition. We also observed malic acid as inactive. The probable reason of its inactivity may be the steric hindrance that in turn hinders the docking phenomenon. Thus, it may seem that molecular interactions of physical or chemical or both types, between maleic acid and the enzyme may be responsible for this unique bioactivity.

In case of tartaric acid, the l (+) tartaric acid showed sprout inhibition activity, while other isomers of tartaric acid failed to inhibit the sprouting. This observation indicates that the centre of interaction of the effective enzyme has a chiral centre.

The most active concentration of maleic as well as l-tartaric acid were found to be 0.2 mg/ml (200 ppm). Sprout suppression was checked only by soaking the potatoes with this solution for 18 h. Upon increasing the concentration, no additional activity was observed. The maleic acid treated tubers did not show sprouting till 40 days after treatment whereas, l-tartaric acid hindered sprouting till 30 days post treatment. Although, sprouting was observed at a significantly low percentage in both cases post that time period. From this investigation it is established that maleic acid and l-tartaric acid are very effective for sprout suppression. Both the acids are extremely cheap and are abundantly available as an industrial raw material^40,41^. The active concentrations for the acids range from 0.2 g to 0.5 g/l/kg of potato. The cost for treatment, therefore, would range 0.004–0.01 INR per kg of potato which is much cheaper as compared to the widely used CIPC^13^. Due to the organic nature and non-toxicity of both acids, these do not have any residual effect. Allelopathic activities of l-tartaric acid have been reported earlier^42^ along with its roles as a basic moiety for synthesis of bioactive molecules and as antimicrobial agents^43^ but its antisprouting activity has not been studied before.

The technique that we implemented for application of these compounds on potatoes is also cost-effective and easy-to-use for small-scale storage and distribution. It has a good promise for handling potatoes, one of the most consumed food crop, in open market after low temperature preservation in developing countries throughout the world.

## Materials and method

### Potato tubers

Potato tubers of the cultivar *Kufri Jyoti* were used for all the experiments as it is widely cultivated variety in India. Potato seeds were purchased from Bidhan Chandra Krishi Vidyalaya, Kalyani, West Bengal, India and cultivated in the farms of our institute. Tubers of medium size (dia. approximately 6–7 cm) and about 4 months old post-harvest, having emerged from dormancy in the month of August–September 2018 were selected and stored at 16 °C until the experiments were set. Potato tubers along with the potato plants were identified by Professor Nanda Dulal Paria, (Former Professor and Renowned Taxonomist, Department of Botany, University of Calcutta, and Former President of Botanical Society of Bengal) and voucher specimen (No. ST-001) has been deposited for keeping record to the Head, Agricultural and Ecological Research Unit, Indian Statistical Institute, Kolkata, India.

All the experiments in our present study complied with institutional guidelines for working on plants and as our model plant is a cultivated species, the work did not involve any violation of IUCN Policy Statement on Research Involving Species at Risk of Extinction and the Convention on the Trade in Endangered Species of Wild Fauna and Flora.

### Sprout inhibition assay

Tubers were washed, air dried and evenly divided in polythene container of capacity 1 l each with 7–8 tubers. The experiments were replicated thrice. Preliminary screening of antisprouting activity was performed using various organic acids (Merck) listed in Table 2. For low solubility of fatty acids and other organic compounds, emulsions of fatty acids (palmitic acid and stearic acid) were made in water with the addition of diethylamine. An experimental control set was prepared using distilled water for all the experiments. For the preliminary screening experiments, a set of three concentrations 0.2, 0.3 and 0.5 mg/ml in water was used for all the tested compounds. The tubers were immersed in the solutions for 18 h in dark at 28 °C. After incubation, the tubers were dried and stored in dark at room temperature (28 °C) in polythene containers until the control set potatoes had all the eyes sprouted. Sprouting percentages were recorded finally after the storage for all the sets. Compounds that could stop sprouting of potatoes even after the control set had sprouted above 95% were considered as a marker for selecting the compounds that show promise against sprouting of potato.

Based on the screening experiments, the compounds that showed significant antisprouting activity were selected for further elaborate experiments. Different concentrations of the effective compounds were tested ranging from 0.1 to 0.3 mg/ml. The treatment of potato tubers were done as described previously. The incubation period for compounds were kept same but the tubers were stored for 42 days and data was recorded at 7 days interval. Experiments were performed with 15 tubers for each treatments and dilutions. All the experiments were replicated thrice.

Sprouting inhibition efficiency was recorded by the following formula

1 $$\mathrm{Sprouting \% }=(\frac{\mathrm{no}.\,\mathrm{ of\, eyes\, sprouted\, in\, each\, tuber}}{\mathrm{total\, no}.\,\mathrm{ of\, eyes\, in\, that\, tuber}}) \times 100$$

### Loss of fresh weight (%)

The fresh weight per tuber was calculated both initially and after the storage time span. The mean loss of fresh wt. for each set was calculated as a percentage^44^.

2 $$\mathrm{Loss\, of\, fresh\, weight \,} \% = (\frac{\mathrm{Initial\, weight}-\mathrm{Final\, weight}}{\mathrm{Initial\, weight}}) \times 100$$

### Reducing sugar content

The total reducing sugar content was estimated using the DNS reagent^45^. Three tubers were taken from each set after the storage period of 42 days. Three tuber pieces of 1 g were cut using a cork borer (1 cm diameter) from each tuber and extracted with 5 ml deionized water. The extracts were centrifuged at 5000 rpm and the clear supernatant was used for the analysis. 2 ml of DNS reagent was added to 1 ml of the extract. Absorbance was taken at 570 nm. Glucose was used as the standard (0.2—2 mg/ml) to make the calibration graph. Amount of reducing sugar was calculated as percentage of the reducing sugar in the tubers. The standard curve of glucose was obtained with the equation: y = 0.2308x + (− 0.0584), R^2^ = 0.9988.

### Total phenolic content

The total phenolic content was measured using the same extract mentioned previously by Folin–Ciocalteau method^46,47^. At first, 100 µl of the extract was mixed with 2 ml of 10% Folin-Ciocalteu reagent and 1.6 ml of 7.5% sodium carbonate (Na_2_CO_3_) followed by incubation for 30 min at room temperature. The color generated was measured in a spectrophotometer (Genesys 180, Thermo Fisher) with absorbance reading at 765 nm. The calibration curve was prepared using a concentration range of 0.03–0.3 mg/ml of gallic acid as standard. Total phenolic content was expressed as mg gallic acid equivalents (GAE)/g fresh wt. Standard curve of gallic acid was obtained by the following equation: y = 0.234x + (− 0.005), R^2^ = 0.990.

### Total protein content

Total protein content was measured using Bradford reagent^48^. Three tubers from each set was selected and a total of three pieces of 1 g were cut using a cork borer (2 cm dia) from each tuber. The tubers were extracted at 4° C using 5 ml of potassium phosphate buffer (50 mM, pH 7.5) containing 2 mM sodium sulphite to reduce enzymatic browning. The extracts were centrifuged at 5000 rpm and the clear supernatant was used for the analysis. 2 ml of Bradford reagent was added to 1 ml of the extract. The absorbance was measured at 595 nm. Bovine Serum Albumin was used as the standard (0.02–0.2 mg/ml) for preparing calibration curve. The total protein content was calculated as the mg BSA equivalent per g fresh wt. The standard curve of BSA was obtained with the equation: y = 3.555x + 0.3911, R^2^ = 0.909.

### Statistical analysis

SPSS 18 software^49^ was used for statistical analysis. The data obtained was put to two way ANOVA test to find out whether the main inhibitory effect and interaction effect of the concentration of tested compounds and duration of storage was statistically significant. The data was also tested using Tukey Post hoc test to compare group means among themselves.

## Conclusion

In our study, we have found that maleic acid has potential sprout suppressant effect on potatoes during storage for a period of 42 days. This compound is active even at 0.2–0.3 mg/ml. l-Tartaric acid has also shown promising results by checking sprouting of potatoes for 30 days. Application of these compounds do not interfere with the quality parameters and also restore moisture content and natural appearance of the potato tubers. These new compounds are cheap, non-toxic and leave no residual effects. They are easy to use and even effective at room temperature. So their implication on commercial scale would be a cost-effective, safe and user friendly both from vendors' and environmental perspectives.

## Supplementary Information

Supplementary Information.

## Data Availability

The data that support the findings of this study are available from the corresponding author upon request.
