# Supplementary material for: Maleic and l-tartaric acids as new anti-sprouting agents for potatoes during storage in comparison to other efficient sprout suppressants
Source: Sci Rep. 2021 Oct 8;11:20029. doi: 10.1038/s41598-021-99187-y (PMC8501061; doi:10.1038/s41598-021-99187-y)
Supplement: Supplementary file 1 — Supplementary Information. [file 41598_2021_99187_MOESM1_ESM.docx]

| Treatments Concentrations (mg/ml) | Sprouting percentages | | | | | | | | | | | | |
| --- | --- | --- | --- | --- | --- | --- | --- | --- | --- | --- | --- | --- | --- |
|  | **Maleic acid** | | | | | |  | **L-tartaric acid** | | | | | |
|  | **7 days** | **14 days** | **21 days** | **28 days** | **35 days** | **42 days** |  | **7 days** | **14 days** | **21 days** | **28 days** | **35 days** | **42 days** |
| 0 | **78.86±19.69** | **81.2±7.71** | **81.9±13.6** | **88.04±11.6** | **91.61±8.12** | **98.07±4.38** |  | **63.3±13.89** | **77.25±2.71** | **82.38±5.66** | **92.82±9.21** | **95.43±2.11** | **98.4±3.93** |
| 0.1 | **23.81±11.93** | **23.9±18.27** | **25.8±7.91** | **26.4±8.21** | **26.8±11.41** | **33.41±15.39** |  | **13.9±14.47** | **21.51±17.2** | **21.82±7.56** | **27.7±6.31** | **31.85±3.66** | **35.92±7.44** |
| 0.2 | **2.5±5.05** | **2.5±5.05** | **3.28±2.92** | **3.71±7.81** | **5.98±7.34** | **7.48±9.53** |  | **4.76±6.81** | **4.81±7.1** | **5.62±9.32** | **10.62±11.71** | **10.68±5.3** | **12.97±12.66** |
| 0.3 | **9.12±7.76** | **9.19±11.42** | **9.34±8.11** | **10.72±11.21** | **10.72± 4.66** | **11.84±6.45** |  | **5.15±7.41** | **5.16±8.24** | **7.63±3.12** | **11.85±9.3** | **15.8±7.77** | **18.59±11.61** |

Table 3 (Supplementary table): Sprouting percentages of the Control as well as Maleic and L-tartaric acid treated potato tubers at 7 days intervals for a storage period of 42 days. The data presented here is mean ± S.D for three replications.
